# Organizational readiness and rehabilitation professionals’ views on integrating telerehabilitation into service delivery and students’ clinical training: A qualitative study

**DOI:** 10.1177/20552076231212314

**Published:** 2023-11-06

**Authors:** Eugene Nizeyimana, Conran Joseph, Quinette A Louw

**Affiliations:** Division of Physiotherapy, Department of Health and Rehabilitation Sciences, 26697 Stellenbosch University, Cape Town, South Africa

**Keywords:** Clinical training, organization, readiness, rehabilitation professionals, service delivery, students, tele-rehabilitation

## Abstract

**Objective:**

To assess the readiness of healthcare institutions that serve as clinical platforms for Stellenbosch University’ rehabilitation students, and to explore the opinions of rehabilitation professionals regarding the integration of telerehabilitation (TR) into service delivery and students clinical training.

**Methods:**

This study employed a qualitative research design and involved the participation of fourteen rehabilitation managers. Semi-structured interviews were conducted using both face-to-face and online platforms. Thematic analysis was employed to analyse the collected data.

**Results:**

The readiness for implementing TR services varies across different dimensions. Facilities faced challenges related to funding for TR equipment and the absence of policies and guidelines, indicating a lack of financial and governance readiness. Rehabilitation professionals demonstrated high attitudinal readiness but low technical readiness due to a lack of knowledge and skills. Rehabilitation students particularly lacked practical experience, confidence, clinical reasoning and decision-making skills further contributing to low technical readiness.

**Conclusion:**

Health care institutions are generally not ready for a successful implementation of TR. To improve the readiness, senior management should actively participate and provide financial support, develop policies, guidelines and training programs for rehabilitation professionals. Educational institutions should incorporate TR program into curricula to prepare students to gain practical experience and familiarity with the use of TR technology for their future clinical practice.

## Introduction

In the last decade, technology has become increasingly used in global health service delivery. Telerehabilitation (TR), which emerged more prominently at the turn of the twenty-first century, has gained popularity as a practice that utilizes information and communication technologies (ICTs) to provide remote clinical rehabilitation services for persons with disabilities and clinical education for healthcare students.^1^ TR is considered a feasible option to enhance access in low -and middle income settings where health systems cannot cope with the demand for services.^2^

In South Africa, the high disease burden and consequent demand of rehabilitation services for disability and functional problems is evident.^3,4^ The integration of TR into service delivery and students’ clinical training holds immense potential to cope with the growing demand for rehabilitation services. Equitable access to rehabilitation services in low resource settings like South Africa is hindered by its vast geography, socioeconomic disparities, constraint resources, poor integration of rehabilitation at all levels of care as rehabilitation is not prioritised by policy makers.^5^ The lack of rehabilitation at primary care and rural regions rehabilitation forces patients to travel long distances for care, which is often not possible due to financial constraints.^5^

TR can potentially be useful in reaching more people who need rehabilitation in a cost-efficient way by utilizing technology. Common ways in which care is delivered include telephonic and video consultations, remote monitoring, and virtual therapy sessions. Using TR, healthcare professionals can provide rehabilitation services directly to patients’ homes or local healthcare facilities where these services are not present. TR has been proposed as a cost efficient medium to extend the reach of services.^6,7^

Traditionally TR has not been integrated into the training of rehabilitation professionals in countries such as South Africa and therefore, professionals may not have the skills to use TR. The COVID 19 pandemic accelerated the use TR as an alternative to deliver services. South African universities are currently exploring the possibilities of integrating TR into the curriculum. As aspiring healthcare professionals, students require exposure and experience in all service modes to ensure they are well-prepared upon graduation. However, health system readiness should be considered for planning the most contextually appropriate way in which TR should be integrated into curricula and used a service delivery mode.

The assessment of organizational readiness is suggested as an important first step before TR is implemented in training and services. The concept of organizational readiness is vital in healthcare because the industry is constantly evolving, with new technologies, treatments, regulations, and best practices emerging regularly.^8^ Healthcare organizations must be prepared to adapt to these changes to provide high-quality care, improve patient outcomes, and remain competitive. Thus, organizational readiness for TR refers to the preparedness and capacity of health care institutions to implement and sustain TR programs effectively.^9^ Factors such as management, technological infrastructure, staff training, policy and guidelines, and financial resources need to be assessed and addressed to ensure a smooth transition and long-term sustainability.^10^ An understanding of TR skills, digital literacy, and knowledge regarding online privacy, ethics and safety is also important prior to the roll-out of TR.^11^ Conducting a readiness assessment to the adoption of TR as an alternative service mode in participating facilities help to identify the challenges that may hinder it's effective implementation and help the organization and rehabilitation professionals to plan and improve the chance of successful implementation.^12^

The view of rehabilitation professionals about TR is also important in shaping the implementation process and influence the acceptance and effectiveness of TR in healthcare settings. Their acceptance and attitudes, perceived benefits, evaluation of clinical efficacy and outcomes, technological competence, and comfort with TR tools are key factors in its successful integration.^13,14^ Understanding their concerns and addressing barriers to adoption is essential for fostering acceptance. Additionally, incorporating their perspectives and experiences on integrating TR into students’ clinical training can help healthcare organizations and educational institutions to optimize the benefits of TR and ensure the delivery of high-quality rehabilitation services.

Furthermore, understanding the current state of organizational readiness and rehabilitation professionals’ views on the integration of TR into service delivery and students’ clinical training can facilitate the design and implementation of tailored TR programs for local context. The aim of this study was to assess the readiness of healthcare institutions that serve as clinical platforms for Stellenbosch rehabilitation students, and to explore the opinions of rehabilitation professionals regarding the integration of TR into service delivery and the clinical training of students. Factors such as technological infrastructure, financial resources, policies and guidelines were explored to determine facilities levels of readiness.

## Methodology

*Ethics:* Ethic approval was received from HREC of Stellenbosch University in January, 2022 (Ref No. N21/11/126) and permission was granted by the Western Cape Department of Health and Wellness (Ref WC_202201_033) prior to data collection, and the authors declare that all procedures contributing to this work comply with the ethical standards of the relevant national and institutional guidelines and with the Helsinki Declaration of 2015.

*Study design:* The study was a qualitative design with an interpretive paradigm to gain deeper understanding of the factors that may influence organization and rehabilitation professionals readiness for integrating TR into service delivery including clinical training for rehabilitation students.

*Setting:* The study was conducted in ten (10) specific clinical sites which provide clinical training opportunities for rehabilitation professional students from Stellenbosch University within the Western Cape Province. The ten participating sites are situated in urban, semi-urban and rural areas of the province. The Western Cape Province is the fourth largest in terms of geographical area and home to approximately 12 million people and boasts the second highest GDP per capita in the country. The province's public health facilities consist of a comprehensive network of hospitals, clinics, and community health centres that cater to the healthcare needs of residents in urban, suburban, and rural areas. These facilities, managed and funded by the provincial government, are designed to serve both insured and uninsured population.

*Population and sampling:* Total population sampling was used to collect the data. Total population sampling is a type of purposive sampling technique. There are about 18 sites from the Department of Health in the Western Cape that accommodate 3^rd^ and 4^th^ year Stellenbosch rehabilitation science students on the clinical platform. All rehabilitation managers working in these 18 sites were eligible and were contacted for voluntary participation. Overall, 14 managers consented and participated in this study, yielding a 78% response rate.

Participating clinical sites included; two tertiary teaching hospitals which offer specialized services and five district hospitals which provide diagnostic, treatment, care, counselling and rehabilitation services ideally on referral from community health centres or clinics. One specialized rehabilitation centre that handles referrals from all levels (tertiary, secondary, district and primary services); and one community centre. Four participating sites are situated in diverse geographical locations, encompassing urban (n = 4), semi-urban (n = 3), and rural areas (n = 3). These sites were purposively chosen because of their involvement in clinical training of Stellenbosch rehabilitation students.

*Data collection instrument:* The lead author (EN) developed an interview guide that included questions covering the main concepts of the study objectives, namely organization readiness and rehabilitation professionals’ readiness. The interview guide questions were adapted from a standardized readiness assessment tool developed by the University of Calgary, focusing on organizational readiness, practitioners’ readiness, and patients’ readiness for the use of Telehealth in clinical and non-clinical settings in New South Wales.^15^ Questions were added to the interview guide to explore rehabilitation professionals’ views and perceptions regarding the integration of TR into clinical training for rehabilitation students. These questions aimed to gather insights on organizational readiness and participants’ perspectives regarding the adoption of TR as a service delivery mechanism and the importance of introducing and exposing rehabilitation students in training on the utility of TR.

**Figure 1.**
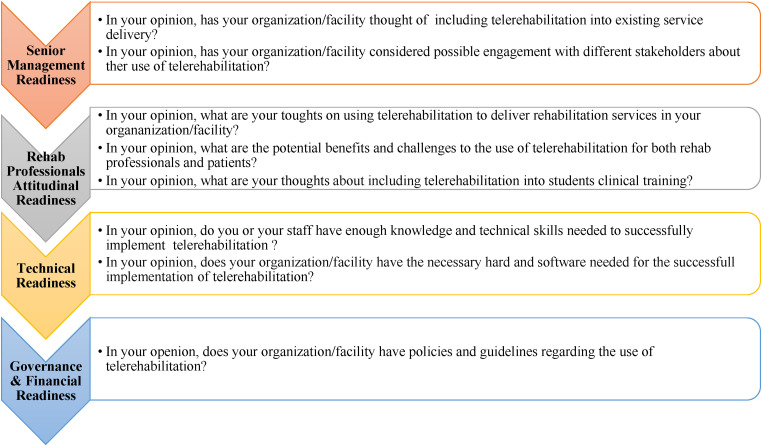
The main themes explored and example of questions asked to explore each theme.

Open-ended questions were used to gather as much information as possible, allowing participants to freely express their views on the factors influencing the adoption of TR and its incorporation into clinical training. To ensure internal validity, the co-author (CJ) reviewed the interview guide to identify any ambiguities that might result in irrelevant questions. Additionally, the interview guide was pilot tested on two participants prior to the main study to further refine and validate its effectiveness. Figure 1 provides an overview of the main themes explored in the interview guide and examples of the questions asked to explore each theme.

## Main themes

### Data collection methods

Data collection began with a pilot study on two participants prior to the main study. The aim of the pilot study was to test the appropriateness of the questions and to provide researchers with some early suggestions on the viability of the research.^16^ The transcripts of pilot study were analysed and the results demonstrated that no changes on the interview guide that was required. Since, no changes were required on the interview guide, the two pilot interview transcripts were included in the analysis of the main study.

*Procedures for both pilot and the main study:* Following the ethical approval, the lead author (EN) sent an invitation (via email) to participants requesting for voluntary participation. The invitation was accompanied by an information sheet clearly explaining the purpose and the ethics concerning the study. The information sheet was also accompanied by a consent form that was signed and returned to the researcher prior to study participation. A semi-structured face to face (n = 9) and online via Microsoft Teams (n = 5) interviews were conducted by the lead author [EN]. The interviewer followed the interview guide although in some cases, the interviewer diverted from the interview guide to obtain the deeper understanding of the point raised, but still related to the overall aim of the study. The interviewer also asked probing questions aimed to direct the interviewees to share as much information as possible in their own words. However, the interviewer avoided influencing the participants’ responses to suit his own perspectives, beliefs, and values. Co-constitution during the interview was done to verify participants’ meaning and researchers understanding by repeating or paraphrasing what the participants have said for assurance. The interview duration ranged between 35 and 40 min.

*Data analysis:* Thematic analysis was employed to analyze data. Thematic analysis is useful method for exploring the perspectives of different research participants.^17^ Interview data were systematically synthetized and grouped into similar themes and categories to produce the meanings and their structure. Both inductive and deductive analysis were used to generate the main themes. The 15- point checklist of criteria for good thematic analysis developed by Braun& Clarke^18^ was used to check the robustness of the data analysis.

*Transcription and data coding:* Guided by six phases of thematic analysis proposed by Braun and Clarke,^19^ the researchers undertook data familiarization by repeated reading and note- taking. A qualitative analysis computer software Atlas.ti.22® was used to code the data. The lead author (EN) developed a codebook using four transcripts and consulted the co- authors (QL & CJ) of the study who are experienced in qualitative analysis to enhance reliability, rigor, and in-depth interrogation of the data.^20^ Each co-author independently analyzed the four transcripts to verify themes, categories and codes identified by the lead author. Each co-author also generated other codes where necessary. All codes generated were compared and discussed by all authors and merged to create a final codebook. The lead author used the final code book to analyze the rest of transcripts.

*Methods of trustworthiness:* The trustworthiness of qualitative research is often questioned by positivists, possibly because their concepts of validity and reliability cannot be addressed in the same way in naturalistic work.^21^ To enhance credibility of the interpreted findings, the interviews were sent back to four selected participants as a form of member checking to seek feedback regarding their accuracy.^22^ The methods employed in data collection, analysis and interpretations of the findings were clearly described to assess transferability. To ensure dependability, research design, its implementation, the operation details of data gathering and reflective appraisal of the transcripts were reported in detail.^21^ The confirmability was achieved through the process audit trail that was established by keeping a detailed record and providing a clear description of the data collection and analysis process including electronic recoding, transcripts, reflective journals and memos, which allows any observer/ non-researcher to trace the course of research step by step via the decision made and the procedures that lead to that decision.^23^

## Results

Participants were 14 rehabilitation managers from four different professions including: Physiotherapists (n = 7); Occupational therapists (n = 4); Speech and language therapists (n = 2) and a professional Nurse (n = 1), working in clinical sites that accommodate Stellenbosch rehabilitation students on the clinical platform. Participating sites are located in the three different geographical locations including: Urban (n = 4), Semi-urban (n = 3) and Rural areas (n = 3). Thirteen out of fourteen participants had more than five years of work experience as rehabilitation managers at the time of data collection. Out of 14 participants, only 1 had a Master's degree while the rest had BSc degrees. Table 1 presents education level, profession, work location and experience information of the study sample.

**Table 1.** Educational, work location and experience information of the study sample.

ID	Qualification	Profession	Location of clinical site	Years of experience
P1	BSc degree	Physiotherapist	Urban	>5 years
P2	BSc degree	Physiotherapist	Semi- urban	>5 years
P3	Master's degree	Physiotherapist	Semi-urban	>5 years
P4	BSc degree	Speech and language therapist	Semi-urban	>5 years
P5	BSc degree	Physiotherapist	Urban	>5 years
P6	BSc degree	Occupational therapist	Urban	>5 years
P7	BSc degree	Speech and language therapist	Urban	<1 year
P8	BSc degree	Physiotherapist	Semi-urban	>5 years
P9	BSc degree	Occupational therapist	Semi-urban	>5 years
P10	BSc degree	Physiotherapist	Urban	>5 years
P11	BSc degree	Occupational therapist	Rural	>5 years
P12	BSc degree	Occupational therapist	Rural	>5 years
P13	BSc degree	Nurse	Rural	>5 years
P14	BSc degree	Physiotherapist	Rural	>5 years

### Themes and categories identified

**Figure 2.**
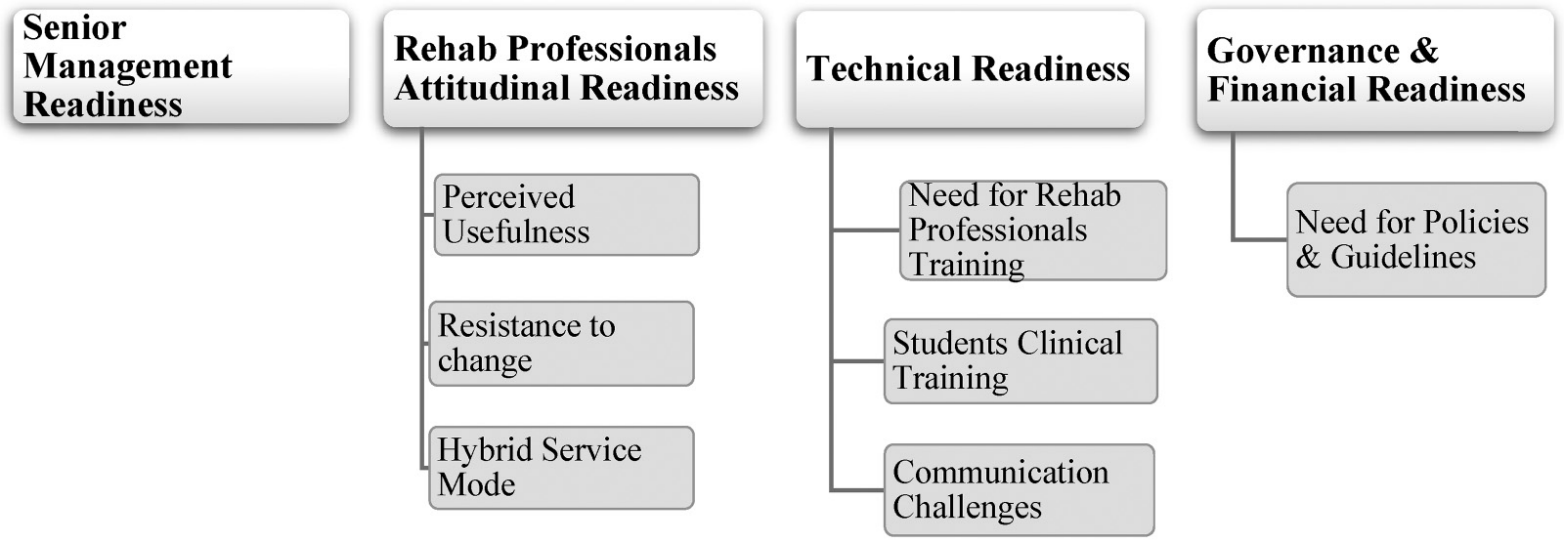
Themes and categories identified.

Thematic analysis identified 4 main themes and 7 categories. The main themes and categories identified are presented in Figure 2 and are further explored below.

#### Theme 1: Senior management readiness

The participants in the study expressed different levels of managerial readiness and involvement in the implementation of TR services within their organizations. Some participants emphasized difficulties they faced due to management's reluctance to allocate funds for necessary TR equipment. The silence of management on the topic indicated a lack of buy-in, and participants believed that if management supported the initiative, it would prioritize funding for the required hardware and software.

*Our management is quiet about telerehabilitation; they are absolutely silent about it. Because if there is buy-in from management, definitely, our hardware and software needed for telerehabilitation will be a priority for them. [P2, Semi-Urban Area]*

This highlights the importance of management support in allocating resources and prioritizing the implementation of TR services. In certain facilities, participants mentioned that top management had not yet discussed TR as viable rehabilitation mode/strategy although the organizations were generally open to partnering with universities and collaborating on such initiatives. This suggests a potential willingness to explore TR, but the level of management's consideration and involvement remained uncertain.

*So, we've not really had a top management discussion until your rehab project. But I mean we are always keen to partner with the university and to work with the university. [P11, Rural Area]*

On the other hand, some participants indicated a level of buy-in from high-level management. They mentioned that the medical superintendent, who is in charge of their section, considers TR as one of their telehealth projects, indicating support and interest from certain individuals in leadership positions.

*I think there is a buy in from them, as I mentioned that the medical superintendent is in charge of our section and telerehabilitation is one of his projects to develop telehealth. So, there is a buy in from the high ups. (P7, Urban area)*

This demonstrates the positive impact of supportive individuals in leadership positions on driving the implementation of TR services. Overall, the participants highlighted the varying degrees of managerial readiness and involvement regarding TR. It is crucial for organizations to address managerial readiness and ensure active involvement to successfully implement TR services. This can be achieved through open discussions, resource allocation, and fostering a culture of support for innovative initiatives like TR.

#### Theme 2: Rehabilitation professionals’ attitudinal readiness

##### Perceived usefulness

Participants expressed a positive attitude towards TR by acknowledging its perceived usefulness and potential benefits. They recognized the convenience and efficacy of TR, emphasizing that it could save time and money for patients while increasing access to rehabilitation services. Participants highlighted that TR eliminates the need for patients to travel long distances to visit rehabilitation centres, thereby eliminating transportation costs and reducing the time they would need to take off work. This aspect is particularly beneficial for patients with limited financial resources or those living in remote areas with limited access to healthcare services.

*The patient have the comfort of sitting in his own home or his workspace to do his assessments and treatments and transport costs is eliminated. [P13, Rural area]*

*So, if patient don't need to come in, then that would help them regarding losing time from work, losing that money, because for some people no work, no pay, so, they lose money from not being at work and they lose money from paying for transport. So, I think that would definitely have a positive financial effect on them. [P14, Rural area]*

Furthermore, participants recognized TR as a valuable backup plan during unforeseen circumstances such as a pandemic. They believed that TR could ensure continuity of care when physical visits are not possible. Participants also mentioned that TR could serve as a valuable support mechanism for patients requiring follow-up care at home, allowing for quick check-ups and interventions tailored to the patient's specific needs

*If another pandemic arises, things might not stop compared to what happened now. Should there be another pandemic, I think in my mind, things will continue with things like this telerehabilitation or technology. Whereas, like in the past things just stopped. [P8, Semi-urban area]*

*But home follow up or telerehabilitation might be a good support mechanism for the patient who may not be able to attend face to face sessions for whatever raison. So, that is access to the rehab. [P11, Urban area]*

##### Resistance to change

Although the majority of participants expressed positive attitudes towards TR and believed in its effectiveness, few of them remain hesitant, showing resistance to change. Their preference for face-to-face therapy and the importance of physical interaction contribute to their resistance. Particularly, experienced health professionals may struggle to accept TR as a valid form of treatment, as they believe it cannot fully replicate the personal connection and tactile feedback they receive during in-person sessions.

*As therapists, we value that interaction and connection with patients, and we enjoy using our hands, especially in acute settings. It would be difficult to change our minds about that connection because it is something we all appreciate about our job. A tele screen is not the same as meeting someone in person. [P5, Urban area]*

*For me, unfortunately, I am a rehab clinician. I have 18 years’ experience. I know what we do, I know what outcomes are, but if there is nothing else, then you have this (Telerehabilitation) and you can go into that. (P8, Semi-urban area)*

Age is identified as a potential barrier to adopting TR for both therapists and patients. Older individuals may struggle with the technology required for remote sessions, including using computers or smartphones. Participants believe that older patients may have difficulty to fully engage in TR sessions due to these technological challenges. Furthermore, the lack of access to necessary devices such as smartphones or computers is seen as a concern. Participants believe that some patients may refuse TR as they do not have the required equipment to participate in remote sessions.

*Yeah, definitely older people are not going to be able to sit in front of a computer and follow what you are trying to do, to guide them through the phone call or whatever. And I am not even that old, but I am also not too keen. [P10, Urban area]*

*I think some of the patients will not accept it (Telerehabilitation), because they don't have access to laptops, the do not have phones, some have got phones but not smart phones. So, they basically will not be able to understand fully I think. [P14, Rural area]*

##### Hybrid services modes

Although participants acknowledged numerous benefits of TR, they also recognized its limitations. They stressed the importance of a cautious approach to implementing TR, understanding that it may not be suitable for all patients or conditions. The participants highlighted the value of hands-on treatment and pointed out that TR has limitations in terms of providing physical contact and assessing certain aspects of a patient's condition. They specifically mentioned the challenges of remotely assessing factors such as facial expressions, range of motion, and end feel, which are crucial in conducting physical assessment, making accurate diagnoses and treatment decisions.

*Because you do need hands on, if you are assessing tone, you need to feel what that tone feels like. If you are assessing range of motion, it is difficult to say “move your arm” can you go a bit further? and especially if you want to feel end feel. What type of end feel does he or she have? Is it bonny end feel? [P9, Semi-urban area]*

Rather than completely replacing face-to-face treatment, the participants argued that TR should be reserved for specific cases and viewed as one tool among many in the toolbox of rehabilitation professionals. They suggested that health professionals should have the option to choose TR when it is suitable for specific patients and conditions.

*I think that telerehabilitation should become a tool of available tools to health professionals that with the right client, they can be able to choose that one.[P1, Urban area]*

Overall, participants perceive TR as a beneficial approach that offers convenience, cost savings, and increased access to rehabilitation services. However, there are reservations about its suitability for all patients and conditions, particularly those requiring hands-on interventions. There is a need for careful consideration of patient characteristics, technological capabilities, and the limitations of remote assessments in determining the appropriateness of TR.

#### Theme 3: Technical readiness

##### Need for rehab professionals’ training

Participants expressed their concerns about the lack of TR training in their university curriculum, as TR is a relatively new approach to therapy. They emphasized the importance of receiving guidance and training from experienced TR practitioners to ensure they provide effective services and execute TR correctly.

*I think what would also be nice is to have someone from the institutions where telerehabilitation have been done successfully to come and train us how to do it, to guide use because some time we are not sure if what we do is correct. (P1, Urban area)*

*Because when we were trained at the university, it was like you are physically working with the patient. So, it would be something new and it would require training. [P12, Rural area]*

The participants recognized the need for comprehensive training, not only for therapists but also for community members and home-based care workers, particularly in rural and semi-rural areas where TR services are provided.

*I think training of all the therapists, training of the community or the home-based care workers because we work in rural and semi- rural, so, I think it would be good for all of us know how to use it (telerehabilitation). [P14, Rural area]*

##### Student clinical training

Furthermore, participants discussed the importance of clinical training for students. They emphasized that students need practical experience, confidence, decision-making skills, and clinical reasoning abilities to effectively deliver TR services. Participants also expressed concerns about students’ ability to handle patient inquiries during TR, as patients often bring up additional complex issues beyond the initial question. They stressed the importance of equipping students with the necessary knowledge and skills to manage such situations effectively.

*Because the students will not be able to guide patients. Because it is one thing to ask a question. Hi, how are you? But what happens on the other side, patients are bringing up a lot of staff. “I have a pressure injury”, “I have UTI”. What will happen then? Who will be equipped to manage those question? (P8, Semi-urban)*

Participants suggested incorporating TR training into the curriculum at the educational level. They believe that introducing TR concepts early on and providing opportunities for students to practice TR during their practical experiences will better prepare them for professional practice in the field.

*I think it is good to include telerehabilitation in the curriculum and I think if you do include it in the curriculum then they must be an opportunity for students to be able to practice that when they do go out and do their practical. [P11, Rural area]*

They also highlighted the importance of training students in telephone etiquette, as proper phone manners are crucial for maintaining a professional therapeutic relationship. Participants cautioned against students becoming too comfortable during TR sessions, as it may compromise their professionalism.

*One thing that I've seen from most of these students, they don't have telephone Ethic. So, that's for me, it is very unprofessional. So, I think that you've got to train people to answer the phone, it happens a lot with my students. They lack telephone ethic. [P14, Rural area]*

*Because for them (students) it might be a very comfortable situation, and the professionalism might slipover because they are so comfortable. (P2, Semi-urban area)*

In summary, the participants emphasized the demand for training and education in TR, not only for therapists but also for managers, healthcare workers, and students. They believe that comprehensive training, starting at the educational level, will ensure the provision of effective TR services and the successful integration of TR into professional practice.

##### Communication challenges

Effective communication is crucial for the successful delivery of therapeutic TR services. However, participants in this study identified several challenges that may hinder communication and impacted the implementation of TR. One major obstacle mentioned is limited access to hardware and software at the facility level . Many facilities lack computers or have outdated ones that do not have essential features like microphones or cameras. This lack of proper equipment significantly hinder the effective communication during online sessions.

*So, we do not have computers geared for telerehabilitation. The computer that I currently have, do not have access to microphone or camera. So, that is a huge challenge. [P4, Semi-urban area]*

Internet connectivity emerged as another significant challenge. Participants highlight the issues such as slow and unreliable internet connections, frequent disconnections, and the need to repeatedly log in due to subpar Wi-Fi signals. These connectivity problems lead to communication delays, interrupt TR sessions, and in some cases making TR sessions longer than usual care consequently leading to frustration for both clinicians and patients.

*So, something that should take 20 min, it is now taking an hour, because of poor and slow internet. The WIFI we have now to log in, every 10 min, it kicks you out. [P2, Semi-urban area]*

Furthermore, constant power disruptions, known as load shedding in South Africa is a major concern raised by participants. Sudden electricity outages during TR sessions may disrupt the communication during assessment and treatment processes and cause inconvenience for both clinicians and patients.

*We have something that I did not mention, the problem with Eskom and we have power cut all the time and that plays a very big role in case you are in the middle of something and boom, electricity is gone. [P4, Semi-Urban area]*

In addition to facility-related challenges, patients’ limited access to suitable devices such as smartphones and computers complicated the delivery of TR services. Patients who lack these devices are unable to fully participate in remote rehabilitation. Moreover, the affordability and accessibility of data posed significant concerns, as patients often have limited data on their phones, further hindering effective communication. Another issue mentioned is the frequent change of phone numbers by patients. This also makes it difficult for rehabilitation professionals to maintain accurate contact and reliable communication with their patients.

*But they (Patients) also do not have access to technology on their side, they do not even have smart phones or computers. So, it is really a challenge. [P7, Urban area]*

*I am just thinking about telephone numbers of the patients, that is one of the major problems we are struggling with the reliable telephone numbers to actually reach that person on. Because it feels to me that sometimes they change the numbers like the way they change underwear's. [P9, Semi-urban area].*

Overall, these barriers collectively impede the effective communication and have a negative impact on the successful implementation of TR services. Addressing these challenges is crucial in order to improve the delivery of TR services.

#### Theme 4: Governance and financial readiness

##### Need for policies and guidelines

While acknowledging the potential benefits of TR, participants expressed concerns that their healthcare settings are not yet ready for its implementation. They highlighted the lack of preparedness in terms of establishing protocols, policies, and guidelines for TR implementation. They emphasize the importance of standardization, clarity, and a unified approach to ensure the safe and effective use of TR methods.

*So, telerehabilitation is something that we have considered but we are not ready for it. We have not developed any guidelines or policies to guide or to govern us with that. (P2, Semi-urban area)*

Privacy and confidentiality emerged as significant concerns, with participants expressing uncertainty about how to protect patient privacy during remote sessions. The presence of other individuals in the patient's environment during sessions is seen as a potential threat to confidentiality.

*It also depends, if this patient has got privacy at home. We do not want to teach the person in one room how to dress when there is five people living in the same room. [P6, Urban area]*

Participants also highlighted concern regarding the billing aspect of TR. The existing billing system seemed not to be equipped to handle the specific requirements of TR services, leading to confusion and uncertainty in charging patients for TR services. Participants emphasized the need for improved clarity, guidance, and education on billing procedures specific to TR services.

*Like we have a billing system, but we did not know how to bill patients, how do we charge these things (telerehabilitation)? [P2, Semi-urban area]*

Standardized billing practices and comprehensive resources are required to ensure accurate charging of patients for TR services.

## Discussion

This study is the first to examine organizational readiness and the perspectives of rehabilitation professionals on integrating TR into service delivery and students clinical training in South Africa. We discuss the findings based on key themes and compare them with previous research to better understand TR readiness, identify trends, and pinpoint areas for improvement.

*Senior management readiness:* The reluctance of management to secure resources for equipment necessary for TR execution was reported in most facilities. This reluctance indicates a lack of financial readiness at facility level. Senior-level administrative support is critical for the success and sustainability of telehealth services in health-care organizations. According to Camden & Silva,^10^ senior level management provide the software and hardware, infrastructure, guidelines and professional development opportunities. They also provide resources including therapeutic skills, technical skills, digital literacy, and knowledge regarding online privacy, ethics and safety. Therefore, senior management should support rehabilitation professionals and provide the necessary financial and governance support to facilitate a successful adoption and implementation of TR.

*Attitudinal Readiness:* The majority of rehabilitation professionals recognized TR usefulness and displayed a positive attitude towards integrating it into existing rehabilitation services and students’ clinical training. Consistent with previous reports,^24–27^ they acknowledged its potential benefits, such as cost and time savings in transportation, increased access to rehabilitation services, convenience, and continuity of care. This positive attitude is a favourable aspect that can be leveraged to drive the implementation of TR successfully.

However, not all rehabilitation professionals that were positive towards the integration of TR into services delivery. A small number of them exhibited resistance to change. This was due to their preference of hands- on treatment and concerns about assessing and treating certain aspects of a patient's condition remotely. These concerns are also consistent with previous reports. The suitability of TR for all types of patients and conditions, particularly the limitations in assessment and treatment options, has been previously reported.^13,24,28–30^ In the review of Rettinger & Kuhn,^24^ practice related issues (Limited examinations, demonstrations, interventions, and assistance.) were the most commonly identified barriers which were reported in about 59% of the reviewed studies. These suggests that healthcare professionals should prioritize comprehensive training and support to address limited examinations, demonstrations and interventions in relation to TR practice.

In order to overcome the forementioned concerns, previous researchers^13,24,31^ have recommended the adoption of TR as a hybrid service mode. This mode of delivery was also suggested by some participants in this study. It is therefore, crucial for all rehabilitation professionals to perceive TR as a flexible and alternative service mode that allows them to have an option to use it when appropriate rather than seeing it as a complete replacement of face to face therapy. Embracing TR as a hybrid service mode will help increase acceptance among those who are resistant to it.

*Technical readiness:* The need for training programs to enhance knowledge and technical skills for rehabilitation professionals was identified indicating the lack of technical readiness. This is also consistent with the previous reports. A study that aimed to explore rehabilitation professionals’ knowledge of TR in Saudi Arabia, reported that about half of the participants lacked knowledge about information technology and cost which led to their limited use of TR system.^32^ According to Nissen and Brockvelt,^33^ education is a critical factor necessary to promote successful implementation of telehealth in clinical practice. Thus, by offering comprehensive training, rehabilitation professionals can develop competence in delivering TR services and addressing the associated challenges effectively.

In terms of students’ clinical training, participants expressed concern about students’ lack of practical experience, clinical reasoning, and decision-making skills to effectively utilize TR in a clinical setting. This further contributed to the students’ lack of technical readiness for TR. According to previous researchers, the absence of formal TR training in the curriculum of most universities limited students awareness about TR applications, which has been identified as significant barrier for students to use TR effectively.^34^ It is crucial for universities to include TR in the curriculum for rehabilitation students and encourage practical training in TR through classroom simulations. By providing students with the opportunity to gain experience and confidence in TR before entering clinical platforms, they can become better prepared TR consultants.

Effective communication during TR service implementation was hindered by the outdated hardware, patients’ device constraints, data affordability, unreliable internet, and frequent power disruptions. This further pointed towards a lack of technical readiness for both service providers and service users. Previous studies have shown that inadequate devices with essential audio capabilities may lead to poor video quality, frequent disconnections. These often cause frustration for service providers and users, and in some cases causing resistance to new technology.^7,35^ It is important that healthcare institutions prioritize bandwidth expansion, especially in remote areas, resolve equipment issues, and upgrade hardware/software to improve TR consultations and interaction quality.

The issue of patients frequently changing phone numbers was another communication issue that posed significant challenges to the implementation of TR in this study. TR heavily relies on effective communication channels for guidance, support, and timely interventions, making these barriers problematic. The solution to this problem is not clear for healthcare providers. However, establishing efficient systems for updating contact information, such as requesting alternate contact details could help address this issue.

*Governance Readiness:* The lack of TR implementation policies, protocols and ethical guidelines across facilities raised a significant concern that indicate a lack of governance readiness. This gap raised issues related to standardization, clarity, safety, and legal aspects of TR use. Protecting patient privacy and confidentiality is a crucial ethical principle that emerged as a significant issue in this study. To safeguard the confidentiality of patient information, researchers have argued that user agreements should be determined within the framework of legal and ethical regulations.^36^

Another issue that indicated a lack of governance readiness was related to the billing of TR services. inadequate bulling systems and lack of guidelines in this matter raised concerns that in some cases lead to confusion and uncertainty among rehabilitation professionals. According to Salmanizadeh et al.,^37^ health care providers should develop and update the guidelines and regulations for telehealth reimbursement.^37^ Therefore, developing policies, guidelines, and standardized billing practices is essential to ensure a successful TR implementation.

### Strength of the study

1. This study included participants from different geographical areas (Urban, semi-urban, and rural areas). Therefore, organizational readiness, perceived benefits and barriers to the integration of TR into service delivery and students clinical training are well represented.
2. The study aimed to recruit rehabilitation managers from 18 clinical sites which accommodate Stellenbosch rehabilitation students on clinical platform and 14 managers (78% response rate) from different clinical sites participated in the study. therefore, these results can be generalizable to the entire targeted population
3. The study targeted rehabilitation managers from 3 different rehabilitation professionals (PT, PT, SLT) and all 3 disciplines were represented in the study. Therefore, the results can also be generalizable in terms of discipline representation.

### Study limitations

This study is not without limitations: Firstly, this study aimed to examine organizational readiness and stakeholders’ views on integrating TR into service delivery and students’ clinical training. However, it only included rehabilitation managers. Although the perceptions of rehabilitation mangers may not differ from the clinicians there was no representation from patient's side. Therefore, the future studies that involve both organization representatives, clinicians and patients are recommended.

Secondary, this study did not include the students views towards TR which would have added the strength to the study.

Thirdly, the study was conducted within one province of South Africa. Although, all 3 rehabilitation professionals (PTs, OTs, SLTs) were represented in this study, their views and perceptions can’t be generalized to all rehabilitation professionals in South Africa.

## Conclusion

The readiness level for implementing TR services for patient care and students clinical training appears to vary across different dimensions but in general, there is a lack of readiness. At facility level, there challenges of management reluctance to provide funding for TR equipment and lack of policies and guidelines indicating the lack of financial and governance readiness. On the other hand, rehabilitation professionals exhibit a high level of attitudinal readiness, as they recognize the usefulness and potential benefits of TR. This positive attitude is a favourable aspect that can be leveraged to drive the implementation of TR. However, they demonstrate a low level of technical readiness due to the lack of knowledge and skills required to effectively implement TR. Rehabilitation students specifically lack practical experience, clinical reasoning, and decision-making skills, further contributing to the none technical readiness on their side. Therefore, there is a need for improving the readiness level for implementation of TR among all facilities. Management should actively be involved and provide financial and governance by providing funds for necessary equipment and developing policies, protocols and guidelines. Enhancing technical readiness through targeted training programs for rehabilitation professionals is crucial. Universities should include TR program into the current curriculum and facilitate rehabilitation students to gain practical experience and familiarity with the use of TR technology for their future clinical practice. Collaboration between health care institutions’ management, healthcare professionals, and educational institutions is essential for creating an enabling environment for TR readiness.
